# Computational Convergence of the Path Integral for Real Dendritic Morphologies

**DOI:** 10.1186/2190-8567-2-11

**Published:** 2012-11-22

**Authors:** Quentin Caudron, Simon R Donnelly, Samuel PC Brand, Yulia Timofeeva

**Affiliations:** 1 Centre for Complexity Science, University of Warwick, Coventry, CV4 7AL, UK; 2 Department of Computer Science, University of Warwick, Coventry, CV4 7AL, UK; 3 Doctoral Training Centre in Neuroinformatics and Computational Neuroscience, University of Edinburgh, Edinburgh, EH8 9AB, UK; 4 Mathematics Institute, University of Warwick, Coventry, CV4 7AL, UK

**Keywords:** Dendrites, Path integral, Sum-over-trips, Morphology, Dendritic computation

## Abstract

Neurons are characterised by a morphological structure unique amongst biological cells, the core of which is the dendritic tree. The vast number of dendritic geometries, combined with heterogeneous properties of the cell membrane, continue to challenge scientists in predicting neuronal input-output relationships, even in the case of sub-threshold dendritic currents. The Green’s function obtained for a given dendritic geometry provides this functional relationship for passive or quasi-active dendrites and can be constructed by a sum-over-trips approach based on a path integral formalism. In this paper, we introduce a number of efficient algorithms for realisation of the sum-over-trips framework and investigate the convergence of these algorithms on different dendritic geometries. We demonstrate that the convergence of the trip sampling methods strongly depends on dendritic morphology as well as the biophysical properties of the cell membrane. For real morphologies, the number of trips to guarantee a small convergence error might become very large and strongly affect computational efficiency. As an alternative, we introduce a highly-efficient matrix method which can be applied to arbitrary branching structures.

## 1 Introduction

 Discovered more than a century ago by Santiago Ramón y Cajal [1], dendrites form the vast majority of the surface area of a neuron, with the dendritic trees of some motoneurons representing up to 97% of total neuronal surface area and 75% of the total neuronal volume [2]. These complex branching structures are responsible for transferring electrical activity between synapses and the soma. As technology evolved, interest in dendrites began to gather momentum, with the invention of sharp micropipette electrodes in the early 1950s allowing intracellular recordings to be made. It was the breakthrough work of Wilfrid Rall [3] on the application of cable theory to dendritic modelling that provided significant insight into the role of dendrites in processing synaptic inputs, the historical perspective of which is summarised in a book by Segev, Rinzel and Shepherd [4]. Recent experimental and theoretical studies reinforce the fact that dendritic morphology and membrane properties play an important role in dendritic integration [5,6]. We refer the reader to the book *Dendrites*[7], devoted exclusively to these formations and revealing their biological complexity at different scales. 

 It has also been known for some time that nonlinear voltage-gated ion channels are present in the dendrites of various types of neurons [8], and many recent dendritic models are constructed by combining the linear (passive) properties of dendrites together with nonlinear (active) dynamics of membrane channels. Although the nonlinear properties of ion channels contribute considerably to neuronal input-output relations, it is important to recognise that the passive properties of dendritic membranes provide the fundamental core for signal filtration and integration, and thus remain an essential component in understanding electrical signalling in dendrites [9]. 

 When branched dendritic fibres are modelled by passive cable equations, the voltage response across the branching structure for any form of applied current can be calculated via a convolution operation, as long as the Green’s function for the given dendritic tree is found. This approach provides an alternative to the compartmental method, based on the discrete spatial approximation of the potential [10,11]. It is not always trivial to construct such a Green’s function for realistic dendritic geometries. Arbitrarily-branching systems are inherently difficult to solve, a fact recognised early by Rall, who proposed a method of mapping the branching structure onto an equivalent cylinder provided that certain geometrical restrictions were satisfied [12]. The work of Koch and Poggio [13], based on the graphical calculus of Butz and Cowan [14], focused on the calculation of the response function for complete dendritic trees in the Laplace (frequency) domain. Later, Rall’s method of equivalent cylinders was extended by releasing the constraints on diameters of individual branches and by constructing the Green’s function, again in the Laplace domain [15,16]. An alternative method for constructing the Green’s function for a branching structure with a shunted soma was proposed by Evans and coauthors [17-19]. In this series of papers, the response function was found in the form of an eigenfunction expansion, which converges particularly rapidly for large times. For smaller times, a Laplace-domain series solution provides better accuracy, agreeing well with an earlier “sum-over-trips” method for constructing the Green’s function directly in the time domain, proposed by Abbott et al. [20]. This sum-over-trips framework is built on a path integral formulation and enables the calculation of the Green’s function on an arbitrary dendritic geometry as a convergent infinite series solution. Cao and Abbott [21] presented an algorithm for a computational realisation of the sum-over-trips approach, based on the division of trips into four classes. They applied this algorithm to a number of sample dendritic trees, the largest of which had 22 branches, in contrast to real dendritic geometries, which might have more than 400 terminals alone [22], with a large variation in branch length. This complexity in neuronal morphologies across different types of neurons is expected to affect the convergence of computational implementations of the sum-over-trips framework. 

In this paper, we introduce and investigate a number of efficient algorithms for calculating the Green’s function on dendritic trees using the sum-over-trips formalism. In Sect. 2, we review the theoretical framework and the four-classes algorithm of Cao and Abbott [21], and introduce alternative algorithms for the sum-over-trips method in Sect. 3. We begin with a modification on the four-classes algorithm aimed at improving its time complexity by developing a formal grammar to derive the trips. Then, a length-priority ordering of the trips using Eppstein’s algorithm [23] for finding the *k* shortest trips on a graph is proposed. We also derive a stochastic approach for sampling trips on the tree based on a Monte-Carlo approach. Finally, a highly-efficient deterministic method for discretised tree structures is described. We assess the convergence of the introduced algorithms on different dendritic geometries in Sect. 4, where we also compare the delay and attenuation of voltage spread on four reconstructed dendritic morphologies. Finally, in Sect. 5, we provide a discussion of our results, as well as possible extensions of this work.

## 2 The Sum-over-trips Framework

We consider a dendritic branching structure with the dynamics of the membrane voltage on a finite branch *i* described by the passive cable equation. An external current $I_{j}(t)$ is injected at a location *y* on branch *j*. The transmembrane voltage across the dendritic tree is then described by the following set of equations:

(1) $$\pi a_{i}C\frac{\partial V_{i}}{\partial t}=\frac{\pi a_{i}^{2}}{4R_{a}}\frac{\partial^{2}V_{i}}{\partial x^{2}}-\frac{\pi a_{i}}{R}V_{i},\;0\leq x\leq L_{i},i\neq j$$

(2) $$\pi a_{j}C\frac{\partial V_{j}}{\partial t}=\frac{\pi a_{j}^{2}}{4R_{a}}\frac{\partial^{2}V_{j}}{\partial x^{2}}-\frac{\pi a_{j}}{R}V_{j}+\delta (x-y)I_{j}(t),\;0\leq x\leq L_{j}.$$

Here, $a_{i}$ is the diameter of branch *i* (measured in μm), $R_{a}$ is the specific cytoplasmic resistivity (in Ω cm), *C* is the specific membrane capacitance (in μF cm^−2^), and *R* is the resistance across one unit area of passive membrane (in Ω cm^2^). Introducing the electrotonic space constant $\lambda_{i}=\sqrt{a_{i}R/(4R_{a})}$, the membrane time constant τ=RC and the diffusion coefficient $D_{i}=\lambda_{i}^{2}/\tau$, Eqs. (1) and (2) can be rewritten as

(3) $$\frac{\partial V_{i}}{\partial t}=D_{i}\frac{\partial^{2}V_{i}}{\partial x^{2}}-\frac{V_{i}}{\tau },\;0\leq x\leq L_{i},i\neq j,$$

(4) $$\frac{\partial V_{j}}{\partial t}=D_{j}\frac{\partial^{2}V_{j}}{\partial x^{2}}-\frac{V_{j}}{\tau }+\frac{1}{\pi a_{j}C}\delta (x-y)I_{j}(t),\;0\leq x\leq L_{j}.$$

In addition to these equations, the appropriate boundary conditions must be specified at all branching nodes and terminals: continuity of the potential across a node and Kirchoff’s law of conservation of current. Continuity of the potential requires that, for all pairs of branches *m* and *n* attached to a node,

$$V_{m}(L_{m},t)=V_{n}(0,t),$$

where the distal end of branch *m* is connected to the proximal end of branch *n*. Conservation of current for the same node imposes

$$\sum_{m}\frac{1}{r_{m}}\frac{\partial V_{m}}{\partial x}{|}_{x=L_{m}}=\sum_{n}\frac{1}{r_{n}}\frac{\partial V_{n}}{\partial x}{|}_{x=0},$$

where $r_{n}=4R_{a}/(\pi a_{n}^{2})$ is the axial resistance on branch *n* (in Ω cm^−1^), and each sum is over all branches connected to this node either with their distal or proximal ends. At individual terminals, we can either impose a closed-end boundary condition,

$$\frac{\partial V_{k}}{\partial x}{|}_{x=L_{k}}=0,$$

or an open-end boundary condition,

$$V_{k}(L_{k},t)=0,$$

where $x=L_{k}$ is a terminal on branch *k*.

When the injected current has the form of a delta pulse, that is, $I_{j}(t)=\delta (t)$, the solution to Eqs. (3) and (4) is the Green’s function $G_{ij}(x,y,t)$ which can be found as

(5) $$G_{ij}(x,y,t)=\frac{1}{\pi a_{j}C}\sum_{\mathrm{trips}}A_{\mathrm{trip}}G_{\infty }(L_{\mathrm{trip}},t),$$

where the sum is over all trips (more formally, graph-theoretic walks), starting at *x* and finishing at *y*, and describes the time-course of the membrane voltage at the location *x* on branch *i* in response to the injected current at the location *y* on branch *j*, where *i* can be taken to equal *j* if desired. The function $G_{\infty }$ takes the form

(6) $$G_{\infty }(L_{\mathrm{trip}},t)=\frac{1}{\sqrt{4\pi tD_{j}}}e^{-{\left(L_{\mathrm{trip}}\right)}^{2}\tau /(4t)}e^{-t/\tau },$$

where $L_{\mathrm{trip}}=L_{\mathrm{trip}}(x/\lambda_{i},y/\lambda_{j})$ is the length of a trip along the tree that starts at point $x/\lambda_{i}$ on branch *i* and ends at point $y/\lambda_{j}$ on branch *j*. Note that the length of each branch needs to be scaled by its own electrotonic space constant before $L_{\mathrm{trip}}$ is calculated for Eq. (6). A constructed trip is allowed to reflect on or pass through any node on the tree an arbitrary number of times. The coefficients $A_{\mathrm{trip}}$ depend on the constructed trip and are determined according to the following rules [20]: 

• From any starting point, $A_{\mathrm{trip}}=1$.

• For every node at which the trip passes from branch *m* to branch *k* where m≠k, $A_{\mathrm{trip}}$ is multiplied by a factor $2p_{k}$.

• For every node at which the trip reflects along on a node back onto the same branch *n*, $A_{\mathrm{trip}}$ is multiplied by a factor $2p_{n}-1$.

• For every terminal, $A_{\mathrm{trip}}$ is multiplied by +1 for the closed-end boundary condition or by −1 for the open-end boundary condition.

 When the electrical properties of the cell membrane are identical for all branches, the factors $p_{k}$ are defined as

(7) $$p_{k}=\frac{a_{k}^{3/2}}{\sum_{m}a_{m}^{3/2}},$$

where the sum is over all branches *m* connected to the node. When the parameters *R* and $R_{a}$ vary from branch to branch, the expression (7) must be modified:

(8) $$p_{k}=\frac{{\left(\lambda_{k}r_{k}\right)}^{-1}}{\sum_{m}{\left(\lambda_{m}r_{m}\right)}^{-1}}.$$

However, note that the sum-over-trips method for constructing the Green’s function in the time domain only works for uniform characteristic time constant *τ* across the entirety of the dendritic tree. The generalisation of this framework to support a quasi-active membrane, instead of a passive membrane, releases this restriction and different cell membrane properties can be chosen on each branch [24]. However, this means that the construction of the Green’s function as an infinite series solution can only be performed in the Laplace domain. 

Knowing the Green’s function for a given dendritic structure allows one to find the voltage response along the entire tree. By finding $G_{ij}(x,y,t)$ for the ordered pair (x,y), the Green’s function $G_{ji}(y,x,t)$ can be found using a simple reciprocity identity:

(9) $$G_{ji}(y,x,t)=\frac{D_{j}r_{j}}{D_{i}r_{i}}G_{ij}(x,y,t).$$

The voltage response can then be found for an arbitrary number of different discrete inputs as a sum of convolution integrals:

(10) $$V_{i}(x,t)=\sum_{j}\int_{0}^{t}G_{ij}(x,x_{j},t-s)I_{j}(s)\;ds,$$

where $x_{j}$ is a location of a stimulus $I_{j}(t)$ on branch *j*.

The Green’s function calculated by Eq. (5) for any branching structure with finite length branches includes an infinite number of terms. It is possible to show that this infinite series solution converges faster than $e^{-k}$, for sufficiently-high *k*, the number of nodes visited by the trip. We demonstrate this in the Appendix for an arbitrary tree with nodes of degree d=3 or less. This generalises Abbott’s convergence analysis [25], where it was shown that, for an infinite binary tree, the sum of coefficients $A_{\mathrm{trip}}$ is O(1) for trips visiting any number of nodes.

### 2.1 Four-Classes Algorithm

 Cao and Abbott [21] introduced an algorithm for constructing the Green’s function using the sum-over-trips method. Their algorithm is based on finding the shortest trips between any two points of measurement *x* and current injection *y* on a tree. Starting from the most direct, shortest trip from *x* to *y*, passing through the minimum number of nodes, four classes of trips are defined by allowing a trip to leave the point *x* in either direction and approach *y* from either direction along their respective branches. These initial trips, therefore, form the first and shortest trips in their respective classes; longer trips are generated incrementally from these. New additional trips can pass the points *x* and *y* any number of times and are allowed to change direction at any node. We will refer to this method as the four-classes algorithm.

**Fig. 1:**
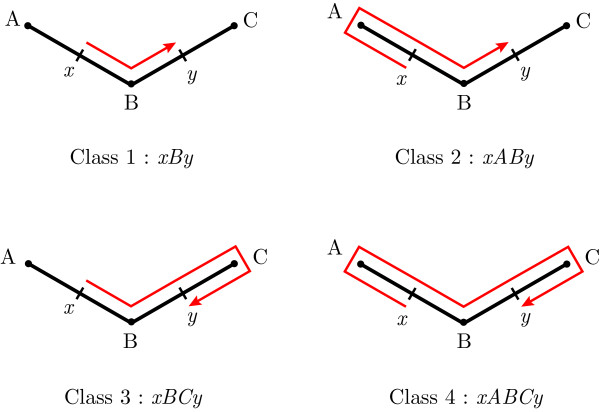
Four classes of trips between two points. *Class 1* is the most direct trip, leaving *x* in the direction of *y* and not going past *y* before finishing (xBy). *Class 2* leaves *x* in the other direction, but finishes when it meets *y* (xABy). *Class 3* moves from *x* towards *y*, but goes past *y* and changes direction immediately after passing it before finishing (xBCy). *Class 4* trips move from *x* first away from point *y* and pass *y* before reflecting on the next node and finishing (xABCy)

Figure 1 shows a model branching structure with two points *x* and *y*, and the four shortest classes of trips between them. Trips are represented as sequences of node identifiers, beginning and ending with *x* and *y* respectively. For example, we denote a trip from *x* to *y* via nodes *A*, *B* and *C* by its full description, xABCy. 

From these main trips, the four-classes algorithm generates all x→y trips by inserting what are described as “excursions” into the trips. If *A* and *B* are adjacent nodes in a tree, then an excursion could be added to the trip xBy to generate the trip xBABy, representing a reflection on node *B* towards *A*, reflecting at the terminal *A* back towards *B*, passing through this node and finally onto point *y*. This process can be iterated indefinitely, generating a trip with two more nodes each time. If this process is applied to every node on every trip with *n* and n+1 nodes, then every trip with n+2 and n+3 nodes will be generated. Thus, from the four shortest x→y trips on the tree, it is possible to construct all trips up to some threshold number of nodes in length explicitly. The lengths and coefficients of these trips can then be calculated from their full trip descriptions, allowing the Green’s function given by Eq. (5) to be approximated.

## 3 Algorithmic Realisations

 Here, we suggest possible modifications to the four-classes algorithm of Cao and Abbott [21] as well as introduce novel alternative algorithms for the sum-over-trips formalism. 

### 3.1 Formal Language Theory Approach

 The four-classes algorithm generates duplicate trips [21], which must then be removed by a binary search through the list of existing trips for every new trip generated, which takes O(klogk) time overall, where *k* is the number of trips constructed. There are two different mechanisms by which duplicate trips are generated, and both mechanisms can be eliminated by applying simple restrictions to the choice of excursions applicable to a trip. As an example of the first, for the tree in Fig. 1, it is possible to generate the trip xBABCBy in two different ways from the shortest Class 1 trip, xBy:

$$\begin{array}{rcl} xBy & \overset{\begin{array}{c} \mathrm{Excursion}\\ B\to BAB \end{array}}{\to } & xBABy\overset{\begin{array}{c} \mathrm{Excursion}\\ B\to BCB \end{array}}{\to }xBABCBy,\\ xBy & \overset{\begin{array}{c} \mathrm{Excursion}\\ B\to BCB \end{array}}{\to } & xBCBy\overset{\begin{array}{c} \mathrm{Excursion}\\ B\to BAB \end{array}}{\to }xBABCBy. \end{array}$$

Due to the fact that the excursion may be added at any step in the trip (at the first or second *B*), the same trip may be generated multiple times. If we insist that excursions cannot be added at any step that precedes the excursion most recently added to the trip, this can be prevented. In the theory of context-free grammars, this is equivalent to requiring a leftmost derivation. We can represent this using a symbol to separate the mutable and immutable parts of the trip: 

The second mechanism by which duplicate trips are produced is the addition of excursions along the same branch, starting from either end. In the structure in Fig. 1, we have both A→A|BA and B→B|AB. Hence, in spite of the leftmost derivation rule, we can generate xABABy in two different ways (brackets added for clarity):

$$\begin{array}{rcl} x|ABy & \to & x(A|BA)By,\\ x|ABy & \to & xA(B|AB)y. \end{array}$$

This problem can be avoided by assigning each branch a direction. If the branch *AB* is given the direction $\vec{BA}$, then the excursion A→A|BA is disallowed. The choice of direction for each branch is unambiguous on acyclic structures: apart from the branch on which *x* is found, each branch must be directed away from *x*. The branch upon which *x* resides is directed away from *y*. This ensures that each node has a sequence of excursions that allow the algorithm to generate trips including it. The allocation of direction to each branch can be performed before the process of generating trips and may coincide with finding the four main classes of trips. These modifications require that the graph be acyclic, since “away from a point” is not generally definable on a graph with cycles. There do exist cyclic graphs for which an unambiguous grammar can generate the language of x→y trips, but these are not relevant to the study of single dendritic trees.

The two presented modifications of the four-classes algorithm are sufficient to prevent the generation of any duplicate trips, without any trips being missed. Together, they provide an unambiguous context-free grammar generating the language of x→y trips.

### 3.2 Length-Priority Method

Since the coefficients $A_{\mathrm{trip}}$ decay at most with $e^{-L_{\mathrm{trip}}}$ (although the number of trips increases with $e^{L_{\mathrm{trip}}}$), the dominating term in the Green’s function (5) is the exponential decay $e^{-L_{\mathrm{trip}}^{2}}$ in $G_{\infty }$. The four-classes algorithm [21] does not generate trips in monotonic order in length, since trips are constructed by adding the same excursion to all four classes of trips. If, for example, a Class 2 trip is significantly longer than its Class 1 counterpart, due to *x* being along a long edge but close to a node, then a longer Class 2 trip will be generated before a potentially shorter Class 1 trip having an additional excursion on a shorter branch. In general, trips are likely to be disordered in length if the branches upon which *x* or *y* reside are substantially longer than at least one other branch on the tree, or if *x* or *y* are much closer to one of their adjacent nodes than to the other.

Here, we propose to realise the sum-over-trips framework by a length-priority method. In this implementation, trips are generated and the corresponding terms $A_{\mathrm{trip}}G_{\infty }(L_{\mathrm{trip}},t)$ are added to the infinite series solution (5) in monotonic order in length $L_{\mathrm{trip}}$. This is achieved by incorporating Eppstein’s algorithm [23] for finding the *k* shortest trips on a graph in O(m+nlogn+k) time, with *n* being the number of nodes and *m* the number of edges on the branching structure.

Both the four-classes algorithm and the improvements described in the language-theoretic approach rely on storing trips explicitly as sequences of nodes. This consumes O(kn) space and time for *k* trips with *n* nodes but allows on-the-fly calculation of coefficients $A_{\mathrm{trip}}$. This is contrary to Eppstein’s algorithm [23], which stores trips using an implicit representation and allows us to find the *k* shortest trips implicitly using only O(1) space and time for each trip. The current implementation, based on Eppstein’s algorithm, requires O(kn) time to calculate coefficients despite the savings on space due to the implicit trip representation. However, Eppstein provides a method for computing any property that can be described by a monoid in O(1) time per trip. Such a description of coefficient calculation exists, and its use would supplement the current O(kn) to O(k) decrease in space requirements with an analogous decrease in time complexity. The savings in space already allow the length-priority method to scale better than the four-classes algorithm.

### 3.3 Monte-Carlo Method

 The path integral formulation of the solution to the cable equation introduced by Abbott et al. [20] is derived via consideration of a Feynman–Kac representation of the solution in terms of random walkers on the dendritic geometry. Hence, it is natural to consider Monte-Carlo approaches to evaluating this path integral. Instead of a length-ordered series solution as provided by the length-priority approach, the Green’s function (5) can be constructed using a stochastic algorithm. The aim of this approach is to sample from trips x→y in such a way that the probabilistically more likely samples coincide with the trips that contribute most to the series solution (5).

To motivate this Monte-Carlo approach, let us consider a linear diffusion equation along an infinite one-dimensional cable,

(11) $$\frac{\partial G}{\partial t}=D\frac{\partial^{2}G}{\partial x^{2}},\;t\in [0,T],$$

satisfying the initial condition G(x,0)=δ(x−y). Analogously, a diffusion process for the state variable $X_{t}$ can be defined by the stochastic equation

(12) $$dX_{t}=\sqrt{2D}\;dW_{t},$$

with the Wiener process $W_{t}$ and the initial condition $X_{0}=y$. It is well known that Eq. (11) is the Kolmogorov equation of the diffusion process (12), that is, the time evolution equation of the probability density for the state of the diffusion (12). On the one hand, solution of (11) via classical numerical or analytical methodology informs the probability density of $X_{t}$; on the other hand, repeated sampling from (12) converges upon the solution G(x,t) of (11). This method of sampling from random walks can also be applied for arbitrary geometries by setting the appropriate boundary conditions at the branching nodes and terminals. Knowing $G_{ij}(x,y,t)$ on a branching structure, we can easily find a solution of the cable equation on this geometry using the relation

$$G_{ij}(x,y,t)=G_{ij}(x,y,t)e^{-t/\tau }.$$

Because the path integral form of the solution is equivalent to the expectation of a function on random walks upon the branching points of the dendritic tree, reduction of the random walk problem from the complete continuous space geometry of the neuron to the discrete topology of the branching points of the neuron gives a considerable efficiency saving to a Monte-Carlo solver. We introduce a parameter $k_{\max}$, the maximum number of discrete hops on nodes for which we wish to calculate the expectation. The maximum number is based upon the effective maximum range of diffusion during the interval [0,t]. Then, we generate a realisation of a random walk on the nodes,

(13) $$\omega =(\omega_{1},\omega_{2},\ldots ,\omega_{k_{\max}}),$$

where each $\omega_{k}$ is a label identifying a particular node. For trips x→y we select $\omega_{1}$ such that it is either of the two nodes adjacent to the branch containing *x*, with equal probability. By indexing a branch between two nodes, $\omega_{k-1}$ and $\omega_{k}$, as the *k*th branch, subsequent steps are performed with the transition probability

(14) $$P(\omega_{k}|\omega_{k-1})=p_{k},\;2\leq k\leq k_{\max},$$

where $p_{k}$ is given by (7). This connects the Monte-Carlo method to the earlier discussed path enumeration methods. Here, we introduce two auxiliary functions, *ϕ* and $\tilde{a}$, of subwalks of *ω*. The first is a function indicating whether a subwalk of *k* steps on a realisation *ω* is a valid trip, and is defined by

$$\phi (y,k,t,\omega )=\left\{ \begin{array}{ll} G_{\infty }(L(y,k,\omega ),t), & \text{if }\omega_{k-1}\text{ and }\omega_{k}\text{ are the nodes adjacent to }y,\\ 0, & \text{otherwise}, \end{array}\right.$$

where *k* is the number of hops on nodes in the subwalk and L(y,k,ω) is the length of the subwalk. The other auxiliary function $\tilde{a}$ is defined as

$$\tilde{a}(k,\omega )=\left\{ \begin{array}{ll} 1, & \text{if }k=1,2,\\ 2, & \text{if }\omega_{k-2}\neq \omega_{k},\\ (2p_{k}-1)/p_{k}, & \text{if }\omega_{k-2}=\omega_{k},\\ 1, & \text{if at a closed terminal (this takes priority)}. \end{array}\right.$$

The relevant function on paths can be defined as a composite of the auxiliary functions described above:

$$\tilde{A}(y,\omega ,t)=\sum_{k=1}^{k_{\max}}2\phi (y,k,t,\omega )\left(\prod_{i=1}^{k}\tilde{a}(i,\omega )\right).$$

The expectation of $\tilde{A}$ with respect to the random walk (14) is equivalent to solving for the path integral, up to some value of $k_{\max}$ at time *t*:

$$\begin{array}{rl} E_{P}\left[\tilde{A}(y,\omega ,t)\right] & =\sum_{\omega }P(\omega )\tilde{A}(y,\omega ,t)\\ & =\sum_{\omega }\sum_{k=1}^{k_{\max}}2P(\omega )\phi (y,k,\omega )\left(\prod_{i=1}^{k}\tilde{a}(i,\omega )\right)\\ & =\sum_{\begin{array}{c} \omega :\\ x\to y\\ \mathrm{at}\;k \end{array}}\sum_{k=1}^{k_{\max}}2P(\omega )G_{\infty }\left(L(y,k,\omega ),t\right)\left(\prod_{i=1}^{k}\tilde{a}(i,\omega )\right)\\ & =\sum_{\begin{array}{c} \mathrm{trips}\\ x\to y \end{array}}A_{\mathrm{trip}}G_{\infty }(L_{\mathrm{trip}},t), \end{array}$$

where P(ω) is the probability of the realisation *ω*, and E denotes the expectation operator. Therefore, the Monte-Carlo strategy is to sample, sequentially or in parallel, the random function $\tilde{A}$ in order to construct this expectation.

### 3.4 Matrix Method

An alternative method of constructing the sum-over-trips series solution is by grouping trips by their lengths:

$$\sum_{\mathrm{trips}}A_{\mathrm{trip}}G_{\infty }(L_{\mathrm{trip}},t)=\sum_{l}G_{\infty }(l,t)\sum_{\begin{array}{c} trips\;with\\ L_{\mathrm{trip}}=l \end{array}}A_{\mathrm{trip}},$$

where the sum over *l* is over all possible trip lengths $L_{\mathrm{trip}}$. On a dendritic tree, discretised as in compartmental models [10] or in a manner similar to the discretisation of the tree into *segments* in NEURON [26], grouping trips according to their lengths allows us to count the number of trips of a given length *l* without having to explicitly construct them.

This method uses a modified directed edge adjacency matrix of the discretised tree in order to compute the sum of coefficients of trips of a given length. It requires all compartments to have the same fixed length Δ*x*, although this restriction can be relaxed in a generalisation presented at the end of this section. The extremities of compartments define the position of nodes; there is a directed edge in both directions between adjacent nodes.

We begin by defining V as the set of nodes and ℰ as the set of directed edges in the discretised tree. Edges are ordered pairs of nodes: e=(u,v)∈E is a directed edge from *u* to *v*, with u,v∈V. For any edge e=(u,v), we denote the reverse edge by $e^{\prime }=(v,u)$. Trips are taken to begin from a point *x* along a starting edge $s=(s_{1},s_{2})$ and end at a point *y* along a goal edge $g=(g_{1},g_{2})$ for s,g∈E. We say that x∈s or $x\in (s_{1},s_{2})$ if *x* resides along edge $s=(s_{1},s_{2})$. Based on the locations of x∈s and y∈g, the orientations of *s* and *g* are defined such that the shortest x→y trip satisfies $x\to s_{2}\to \cdots \to g_{1}\to y$. Therefore, the shortest x→y trip always starts on edge *s*, that is, in the $s_{1}\to s_{2}$ direction, and approaches *y* along the edge *g*, in the $g_{1}\to g_{2}$ direction. This is equivalent to a Class 1 trip; Class 2 trips leave *x* along the $s^{\prime }=(s_{2},s_{1})$ edge, arrive at y∈g; Class 3 trips go from x∈s to $y\in g^{\prime }$; Class 4 trips, finally, go from $x\in s^{\prime }$ to $y\in g^{\prime }$. The locations of the points x∈s and y∈g along their respective edges are given as a fraction of the branch length such that xΔx denotes the distance from *x* to $s_{2}$ and yΔx is the distance between node $g_{1}$ and point *y*. We distinguish between *k*, the number of edges travelled in a particular trip, from the length of the trip $L_{\mathrm{trip}}$. Because *x* and *y* reside along their respective edges, the total length of a trip that travels along *k* edges is less than if the full distance along *k* edges had been travelled. That is, $L_{\mathrm{trip}}<k\Delta x$ for any combination of *x*, *y* and for all *k*.

The aim of the matrix method is to group all trips starting on a given edge and finishing on a target edge by their lengths, $L_{\mathrm{trip}}$, and calculate the sum of the coefficients $A_{\mathrm{trip}}$ of those trips for each particular group instead of calculating coefficients individually for each trip. The sums of coefficients are computed simultaneously for trips ending on all edges, starting at a point $x\in (s_{1},s_{2})$, allowing us to compute $G_{ij}$ for all *j*, for all *x* on edge *i* and for all *y* on any edge, in a single run of the calculation. We define the coefficients function $c_{k}^{s}:E\to R$, s∈E, as the sum of all coefficients $A_{\mathrm{trip}}$ which begin at point x∈s and travel over *k* edges, finishing on a given edge *g*:

$$c_{k}^{s}(g)=\sum_{\begin{array}{c} \mathrm{trips}\\ x\to \cdots \to y\\ in\;k\;\mathrm{jumps} \end{array}}A_{\mathrm{trip}},\;x\in s,y\in g.$$

Because the set of edges ℰ is both ordered and finite, then $c_{k}^{s}=(c_{k}^{s}(e_{1}),\ldots ,c_{k}^{s}(e_{\left|E\right|}))\in R^{\left|E\right|}$ can be thought of as a vector, where $e_{i}\in E$ for i=1,…,|E|. The *i*th element of the vector $c_{k}^{s}$ corresponds to the sum of coefficients $A_{\mathrm{trip}}$ for all trips originating at *x* on *s* and ending along the *i*th edge $e_{i}$, having travelled over *k* edges. The vector $c_{1}^{s}$ consists mostly of zeros, with a one only in the entry corresponding to the edge *s*, as the coefficient of moving in this direction remains 1, while all other moves are invalid by travelling over only one edge, and hence have coefficient 0.

We can now define a matrix $Q\in R^{E\times E}$ such that

(15) $$Q^{k}c_{1}=c_{k+1}.$$

*Q* is a modified form of the edge-adjacency matrix, where instead of containing ones to denote edge adjacency and zero otherwise, it contains the coefficient taken in moving from one edge to another. The entries of *Q* can be computed based on the morphology of the graph. If the *j*th entry corresponds to edge (u,v) and the *i*th entry to edge (v,w), then the entry $Q_{ji}$ is the coefficient taken when moving from branch (u,v) to (v,w). In the general case, these numerical values must be determined for each entry. However, in the simplified case where the radii on all branches are equal and all nodes have degree d=1 or d=3, the matrix *Q* can be constructed according to

(16) $$Q_{ji}=\left\{ \begin{array}{ll} -\frac{1}{3}, & \text{if }j=(u,v)\text{ and }i=(v,u)\text{, where }v\text{ is a node of degree }d=3,\\ 1, & \text{if }j=(u,v)\text{ and }i=(v,u)\text{, where }v\text{ is a closed terminal (}d=1\text{)},\\ \frac{2}{3}, & \text{if }j=(u,v)\text{ and }i=(v,w)\text{, where }u\neq w,\\ 0, & \text{otherwise}. \end{array}\right.$$

Note that the above rules apply to the transpose of $Q_{ij}$.

Thus, knowing the matrix *Q* from the dendritic geometry and the vector $c_{1}^{s}$ from the starting edge *s*, it is possible to construct the sum of $c_{k}^{s}(g)$ terms, for all $k<k_{\max}$, equal to the sum of coefficients for all trips travelling up to $k_{\max}$ edges, from x∈s to y∈g. However, by considering trips moving from *x* in one direction only and arriving at *y* from only one direction, we have calculated the coefficients of just Class 1 trips. In order to find coefficients for the remaining three classes, we must also compute $c_{k}^{s^{\prime }}(g)$, $c_{k}^{s}(g^{\prime })$ and $c_{k}^{s^{\prime }}(g^{\prime })$. These can be found in the same way as above. Using (15), the Green’s function in (5) can therefore be written as

(17) $$\begin{array}{rl} G_{ij}(x,y,t)= & \sum_{\begin{array}{c} \mathrm{trips}\\ x\;\mathrm{to}\;y \end{array}}A_{\mathrm{trip}}G_{\infty }(L_{\mathrm{trip}},t)\\ = & \sum_{k=1}^{k_{\max}}[{\left(Q^{k-1}c_{1}^{s}\right)}_{g}G_{\infty }\left(L_{1}(k),t\right)\\ & +{\left(Q^{k-1}c_{1}^{s^{\prime }}\right)}_{g}G_{\infty }\left(L_{2}(k),t\right)\\ & +{\left(Q^{k-1}c_{1}^{s}\right)}_{g^{\prime }}G_{\infty }\left(L_{3}(k),t\right)\\ & +{\left(Q^{k-1}c_{1}^{s^{\prime }}\right)}_{g^{\prime }}G_{\infty }\left(L_{4}(k),t\right)], \end{array}$$

where ${\left(Qc_{1}^{s}\right)}_{g}$ is the *g*th element of the matrix-vector product of *Q* and $c_{1}^{s}$. Lengths $L_{1},\ldots ,L_{4}$ are the lengths of Class 1 to Class 4 trips, respectively, and are defined as

$$\begin{array}{rl} L_{1}(k) & =\Delta x\left(2(k-1)+x+y\right),\\ L_{2}(k) & =\Delta x(2k-x+y),\\ L_{3}(k) & =\Delta x(2k+x-y),\\ L_{4}(k) & =\Delta x\left(2(k+1)-x-y\right). \end{array}$$

By selecting a small Δ*x*, branches may be approximated by a discretisation using an integer number of edges of length Δ*x*. As in compartmental models, this allows the full morphology of the dendritic tree to be approximated, in a trade-off between high speed (large Δ*x*) and accuracy (small Δ*x*). As Δx→0, however, this approach tends to the computational complexity of naively integrating the cable equation using numerical methods. As in numerical simulations, where reducing Δ*x* in order to increase accuracy brings about a necessary and associated change in Δ*t*, the same is true of the matrix method: selecting a small Δ*x* and hence increasing |E|, implies that $k_{\max}$ must be increased.

This algorithm can be generalised to accept several discrete edge lengths $\Delta x_{1},\ldots ,\Delta x_{n}$, at an exponential cost in the number of different lengths *n*, allowing “caricature” neurons to be constructed from a small number of different edge lengths. Our description of this method is focused on the case where *x* and *y* are located on different branches. For computations where *x* and *y* are required to exist on the same edge, the edges can be discretised such that *x* and *y* appear on different segments. In all cases with bounded node degree, *Q* is a sparse matrix with only a few entries per row, and O(|E|) entries altogether, making the complexity for the calculation of all coefficients $O(|E|k_{\max})$ by using highly-efficient sparse linear algebra algorithms.

#### 3.4.1 Example calculation

**Fig. 2:**
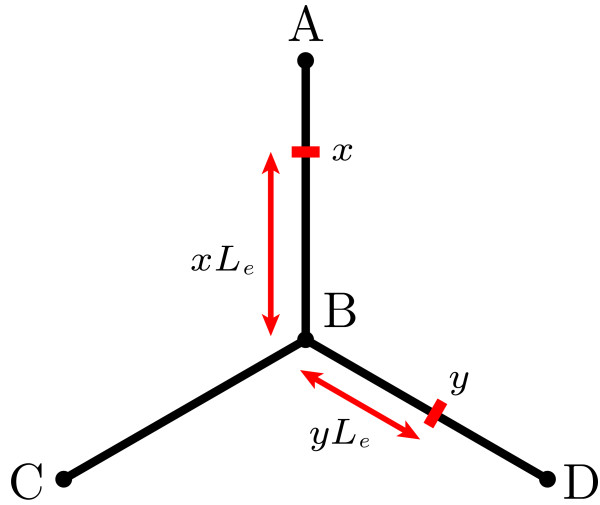
A dendritic structure used in the example calculation for the matrix method

Here, we demonstrate an example realisation of the matrix method for a dendritic structure of three branches of equal length Δ*x*, shown in Fig. 2. In this symmetrical case, the matrix *Q* is very small and can be constructed by hand. We place the point of measurement *x* along edge s=(A,B) and the point of current injection *y* along g=(B,D). 

We begin by ordering the edge pairs as follows: (A,B), (B,A), (B,C), (C,B), (B,D), (D,B). Based on this ordered set, we can obtain two coefficients vectors $c_{1}$, one for trips that begin at *x* and move towards *B*, denoted $c_{1}^{s}={\left(1\;0\;0\;0\;0\;0\right)}^{T}$, and another for trips moving from *x* towards node *A*, denoted $c_{1}^{s^{\prime }}={\left(0\;1\;0\;0\;0\;0\right)}^{T}$. Using the rules described in (16), we can construct the matrix *Q* for our dendritic structure as follows:

$$Q=(\begin{array}{cccccc} 0 & 1 & 0 & 0 & 0 & 0\\ -\frac{1}{3} & 0 & 0 & \frac{2}{3} & 0 & \frac{2}{3}\\ \frac{2}{3} & 0 & 0 & -\frac{1}{3} & 0 & \frac{2}{3}\\ 0 & 0 & 1 & 0 & 0 & 0\\ \frac{2}{3} & 0 & 0 & \frac{2}{3} & 0 & -\frac{1}{3}\\ 0 & 0 & 0 & 0 & 1 & 0 \end{array}).$$

Note that all rows and columns sum to 1. Knowing this matrix *Q* and breaking the trips into four main classes, it is straightforward to find the complete Green’s function:

(18) $$\begin{array}{rl} G_{sg}(x,y,t)= & \sum_{k=1}^{k_{\max}}{\left(Q^{k-1}c_{1}^{s}\right)}_{g}G_{\infty }\left(\Delta x(2n+x+y),t\right)\\ & +\sum_{k=1}^{k_{\max}}{\left(Q^{k-1}c_{1}^{s^{\prime }}\right)}_{g}G_{\infty }\left(\Delta x(2n+2-x+y),t\right)\\ & +\sum_{k=1}^{k_{\max}}{\left(Q^{k-1}c_{1}^{s}\right)}_{g^{\prime }}G_{\infty }\left(\Delta x(2n+2+x-y),t\right)\\ & +\sum_{k=1}^{k_{\max}}{\left(Q^{k-1}c_{1}^{s^{\prime }}\right)}_{g^{\prime }}G_{\infty }\left(\Delta x(2n+4-x-y),t\right). \end{array}$$

## 4 Convergence of Methods

**Fig. 3:**
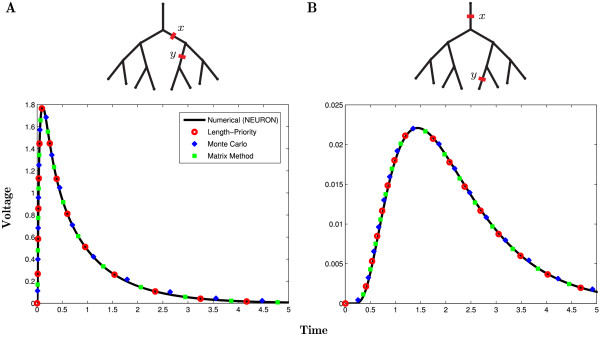
The Green’s function constructed by a number of methods. The voltage traces show the analytical solutions $G_{ij}(x,y,t)$ for fixed *x* and *y* on a binary tree for the length-priority method (*red circles*), the Monte-Carlo method (*blue diamonds*) and the matrix method with $k_{\max}=100$ (*green squares*) superimposed on NEURON’s numerical solution (*black line*). Parameter set A (Table 1) is used in these computations

We first validate the computational implementations of our algorithms by constructing the Green’s function $G_{ij}(x,y,t)$ on a small binary tree. Two profiles of the response function obtained by the length-priority, the Monte-Carlo and the matrix methods are shown in Fig. 3 and compared to a numerical simulation computed by the software package NEURON [26]. These plots demonstrate an excellent agreement between a number of approaches for obtaining the Green’s function and the numerical solution, with a slightly worse performance of the Monte-Carlo method for larger times. 

The computational convergence of any algorithm impacts both the accuracy of its results and the speed at which these results are obtained. While the series solution to the Green’s function (5) is proven to converge for a sufficiently-high number of terms (see the Appendix), this assumes optimal ordering of terms in the solution. Because the coefficients $A_{\mathrm{trip}}$ are impossible to compute until a trip is constructed, generating terms for the series solution in descending order of magnitude is inherently difficult. The four-classes, length-priority and matrix methods generate trips in order of increasing $L_{\mathrm{trip}}$, with the aim of ordering trips by their $G_{\infty }(L_{\mathrm{trip}},t)$ terms, which decreases monotonically in the length of the trip. The Monte-Carlo method uses a stochastic method to order trips by their probabilities, with more likely trips contributing more to (5). However, none of these approaches order the trips optimally, and hence their accuracy relies not on the theoretical convergence of the mathematical method, but on the computational convergence of the algorithm that implements it.

**Fig. 4:**
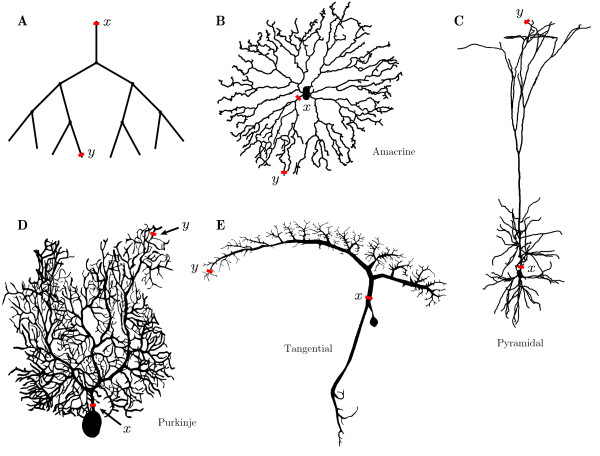
Neuronal structures used in construction of the Green’s function. **A**: a binary tree, **B**: a rabbit amacrine cell [28], **C**: a rat pyramidal cell [29], **D**: a rat Purkinje cell [30], and **E**: a blowfly tangential cell [31]

To assess the computational convergence of the algorithms, Green’s function solutions were constructed on a number of branching geometries shown in Fig. 4. In addition to a binary tree with the topology in Fig. 4A, the algorithms were also applied to four real neuronal reconstructions obtained from the NeuroMorpho Database [27] in .swc format and shown in Figs. 4B–E. These files use a sequence of nodes with precise radii and three-dimensional locations to describe the location of the soma and the paths taken by the axon and each dendrite. A dendrite’s path can be described using as many nodes as necessary to accurately reflect the spatial jitter and variation in radius of its path. Because radii are described at nodes, edges between two nodes of different radius taper. The sum-over-trips formalism requires constant diameter along edges, but allows discontinuous jumps in the diameters at nodes. Hence, edge diameter was defined as the average of the diameters of adjacent nodes. This allows full dendritic branches to be represented as a sequence of uniform cylinders of arbitrary length and with abrupt changes in diameters at nodes. 

We used the following normalised $L^{1}$ error as a measure of convergence:

$$\varepsilon =\frac{1}{V_{N}}\int_{0}^{T}\left|G_{ij}(x,y,t)-V^{*}(x,y,t)\right|\;dt,$$

where *T* is the final simulation time, $V^{*}(x,y,t)$ is NEURON’s numerical solution to very high accuracy and $V_{N}=\int_{0}^{T}V^{*}(x,y,t)\;dt$ is the integral of the accurate NEURON solution. This convergence measure is therefore relative to the amplitude of the “real” solution, and thus errors *ε* are comparable between different neuronal types.

**Fig. 5:**
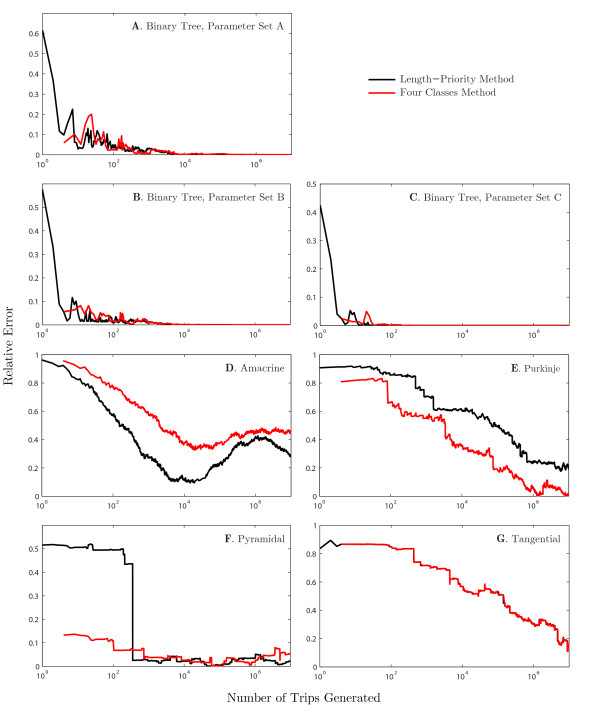
Convergence of the four-classes and length-priority methods for a number of dendritic morphologies. The relative error *ε* of the approximation of $G_{ij}(x,y,t)$ is shown as a function of the number of trips in the sum-over-trips framework for injection at *y* and measurement at *x* on the dendritic trees in Fig. 4. Membrane parameters for real dendritic morphologies: $C=1{\text{ μF cm}}^{-2}$, $R=3\text{,}000\;\Omega {\text{ cm}}^{2}$ and $R_{a}=100\;\Omega \text{ cm}$. Note that the four-classes method always begins with four trips, and each step in the algorithm adds a further trip of each class

Figure 5 shows the convergence of the four-classes and of the length-priority methods as a function of the number of trips on five geometries from Fig. 4. Three sets of parameters given in Table 1 were considered for the binary tree, and the relative errors *ε* for each case are demonstrated in Figs. 5A–C. These plots illustrate fairly uniform convergence, in which both methods offer similar accuracies and rates of convergence. Of the two trees with biophysically realistic parameters, the binary tree with the longer branches (parameter set C) converges faster, as is expected on structures with longer trips in each Class. This is reflected in Table 2, which shows the number of trips required on the binary tree to remain under a given error threshold for different parameter sets. Moreover, a binary tree with non-dimensionalised parameters (parameter set A) requires noticeably many more trips for desired accuracy in comparison to the same tree with biophysically realistic parameters. 

**Table 1:** Parameter sets of the binary tree in Fig. 4A

	Parameter set A	Parameter set B	Parameter set C
Branch length *L*	0.3	50 μm	100 μm
Branch diameter *a*	0.05	1 μm	1 μm
Diffusion coefficient *D*	1	$2.5\times 10^{4}{\text{ μm}}^{2}{\text{ ms}}^{-1}$	$2.5\times 10^{4}{\text{ μm}}^{2}{\text{ ms}}^{-1}$
Membrane time constant *τ*	1	3.3 ms	3.3 ms
Membrane capacitance *C*	1	$1{\text{ μF cm}}^{-2}$	$1{\text{ μF cm}}^{-2}$

**Table 2:** Length-priority method on a binary tree: number of trips required for a given accuracy

Binary tree	Relative error threshold *ε*			
	0.1	0.05	0.01	0.001
Parameter set A	3,240	8,750	1,820,000	>5 × 10^7^
Parameter set B	825	2,600	129,000	>5 × 10^7^
Parameter set C	22	65	815	7,700

Figures 5D–G show *ε* for the structures in Figs. 4B–E respectively. They demonstrate that convergence is non-trivial on complex branching structures. Figure 5D shows that the length-priority method makes consistently less error on the amacrine cell geometry, in contrast to the convergence of the Purkinje cell, shown in Fig. 5E, where the four-classes method generates less error for all numbers of trips. Both of these show strongly irregular convergence and high-amplitude oscillation in the errors *ε* in the amacrine cell. For both methods, the Purkinje cell shows a plateau in error for Green’s functions with few trips, indicating that either these trips are of small magnitude or that their voltage traces alternate between undershooting or overshooting the correct solution between subsequent trips. This indicates that neither the length-priority or the four-classes methods are good heuristics for ordering terms in the Green’s function. This is further hinted at by the oscillating property of the error, which implies that there are regions where trips that increase the error are more frequent than trips that reduce it.

The pyramidal cell’s convergence shows very discontinuous behaviour (Fig. 5F), particularly in the length-priority method. The large jump in error when approximately 350 trips are included in the Green’s function was found to be caused by the first and shortest Class 2 trip included thus far, with all prior trips belonging to Class 1. This behaviour is likely to arise if there exist very short branches along the shortest and most direct x→y trip, and thus many Class 1 trips are generated first, being shorter than the first Class 2 trip. Whilst one of the motivating reasons for considering a length-priority approach was to generate trips fully by length order, this heuristic makes no attempt to include the coefficient $A_{\mathrm{trip}}$ in its ordering. This is an example of a pathologically large change in the coefficients value for a Class 2 trip which contributes a very significant amount to the Green’s function. The four-classes approach, which enforces generation of trips of all four classes at every added excursion, does not show such a drastic drop in error. However, the error plot is still very discontinuous, and this may be a characteristic of situations as we have just described, where points *x* and *y* are placed on branches having a very different length to those on the most direct x→y trip, or when these points are placed very close to a node. Whether injection and measurement points are located on branches that are significantly longer or shorter than those along the shortest x→y trip, both the four-classes and the length-priority methods will generate trips in an “unnatural” order, subsampling the trips where current will spread the most, but oversampling in areas of the tree with very short branches. This pathological feature may not be inherently present in the real neuronal morphology, but may have been created during digital reconstruction from slice image data if, for example, a change of radius were found along the branch. Therefore, this pathology may not be representative of the neuronal geometry, but becomes a function of the reconstruction.

The tangential cell’s convergence, shown in Fig. 5G, shows almost identical errors for both the four-classes and the length-priority methods, indicating that trips are generated in a similar order regardless of method. Contrary to the example with the pyramidal cell, this behaviour is likely to occur when *x* and *y* are placed on branches that are significantly shorter than those that arise on the shortest x→y path, such that the length-priority method returns trips of Class 1, 2, 3 and 4 in sequential order, as these increases in length are shorter than adding an excursion along the direct x→y trip.

Our results clearly indicate that the convergence of the realisation of the sum-over-trips framework by either the four-classes or the length-priority method strongly depends on a dendritic geometry. For real morphologies, the number of trips required quickly becomes very large to the point where guaranteeing convergence to within some small error threshold may become computationally expensive.

**Fig. 6:**
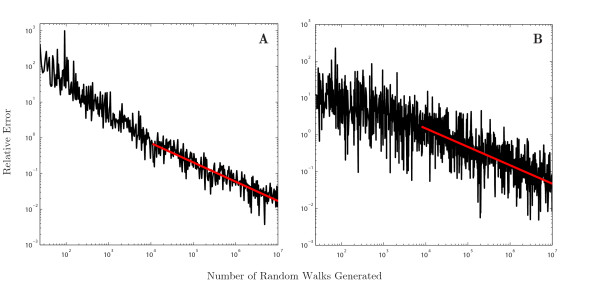
Convergence of the Monte-Carlo method for a binary tree. The relative error *ε* is shown as a function of the number of random walk realisations generated, *k*. **A**: error generated on the binary tree in Fig. 4A with parameter set A. The *red line* shows a fit for $\varepsilon ∼k^{-0.54}$. **B**: the convergence error on a binary tree of depth 16 (65,536 nodes). The *red line* demonstrates a fit for $\varepsilon ∼k^{-0.5}$, the typical rate of convergence of a Monte-Carlo integration

The convergence of the Monte-Carlo method is shown in Fig. 6 for the binary tree in Fig. 4A with parameter set A and for a larger binary tree of depth 16 with the same parameters. Boundary effects can be seen on the smaller tree, where the convergence rate is slightly faster than that of a typical Monte-Carlo integration observed here for a larger tree. It is worth noting that the *x*-axis on this plot shows the number of random walks generated; however, due to the number of sub-trips extracted from each random walk, the number of terms contributing to the Green’s function can potentially be significantly different. The graphs show that the Monte-Carlo method is very slow to converge, although the method is much more predictable in its convergence despite the noise. As expected, therefore, the Monte-Carlo method generates trips that are more “naturally” ordered, and hence convergence is much more monotonic. Despite this improved ordering of terms in the series solution, the Monte-Carlo approach remains computationally intensive and very slow to converge with increased number of trips. 

**Fig. 7:**
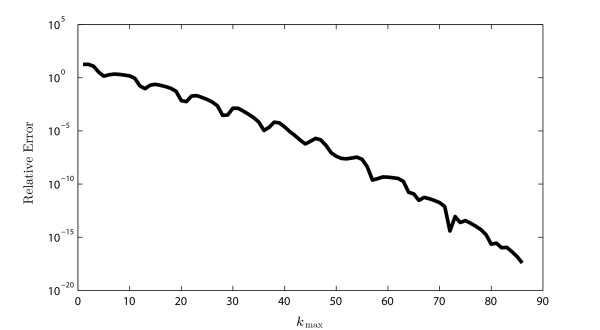
Convergence of the matrix method. The relative error *ε* is shown as a function of the maximal number of edges travelled in the trips, $k_{\max}$, for a binary tree in Fig. 4A

Finally, the convergence of the matrix method on a binary tree is shown in Fig. 7. This algorithm converges extremely quickly to within very small error tolerances as a function of $k_{\max}$, the maximum number of edges covered by trips generated. The values of the product of $A_{\mathrm{trip}}$ coefficients obtained by this method remain O(1) for all $k_{\max}$ which agrees with the proof in [25]. Because the algorithm is based on simple matrix-vector multiplication, where the matrix is |E|×|E| in size, the computation of the Green’s function for small trees such as the binary trees used here to within $\varepsilon =10^{-15}$ only takes a fraction of a second. On more complex trees, such as Purkinje cells, this becomes more expensive, although computing $G_{ij}(x,y,t)$ for the whole tree remains computationally preferable to the use of brute-force simulators. Using the reciprocal rule (9), this is possible in |E| applications of the algorithm. This compares favourably with the length-priority and the four-classes methods, which require |E|(|E|+1)/2 applications of the algorithm, and with NEURON, which would require ${\left|E\right|}^{2}$ simulations, and this would only provide solutions for a single point *y* on each edge. In addition, the sparseness of the matrix *Q* means that coefficient calculation up to $k_{\max}$ only takes $O(|E|k_{\max})$ time, and so the method scales linearly with the number of branches on the tree. 

### 4.1 Structural-Electrotonic Properties

The Green’s function $G_{ij}(x,y,t)$ constructed for a given dendritic geometry provides a measure of the transfer impedance between the input location *y* on branch *j* and the point of measurement *x* on branch *i*. Assessing whether the neuronal geometry significantly impacts this input-output relation is a step towards answering questions regarding the structure-function relationship behind the enormous natural variation in dendritic morphologies. Using the measures introduced by Zador et al. [32], the propagation delay and the log-attenuation, we analyse the transfer of the response signal in four reconstructed cells in Fig. 4. For a pair of points (x,y) along the tree, we reintroduce the propagation delay $P_{xy}$ as a measure of the impact of the tree’s electrotonic structure on the timing of signals, defined as

$$P_{xy}={\hat{t}}_{x}-{\hat{t}}_{y}.$$

Here, ${\hat{t}}_{x}$ and ${\hat{t}}_{y}$ are the centroids of the two corresponding voltage transients $G_{x}(t)=G_{ij}(x,y,t)$ and $G_{y}(t)=G_{jj}(y,y,t)$ respectively:

$${\hat{t}}_{x}=\frac{\int_{0}^{\infty }tG_{x}(t)\;dt}{\int_{0}^{\infty }G_{x}(t)\;dt}\;\text{and}\;{\hat{t}}_{y}=\frac{\int_{0}^{\infty }tG_{y}(t)\;dt}{\int_{0}^{\infty }G_{y}(t)\;dt}.$$

This delay measure admits an additive property such that $P_{xy}=P_{xz}+P_{zy}$ for a point *z* between *x* and *y*. The log-attenuation of the response signal between a pair of points (x,y) is computed as $L_{xy}=\log A_{xy}>0$, where

(19) $$A_{xy}=\frac{\int_{0}^{\infty }G_{y}(t)\;dt}{\int_{0}^{\infty }G_{x}(t)\;dt}\geq 1.$$

It acts as a measure of the amount a transient signal’s amplitude diminishes as it travels between two points. $L_{xy}$ is also additive for a point *z* between *x* and *y*, that is, $L_{xy}=L_{xz}+L_{zy}$.

**Fig. 8:**
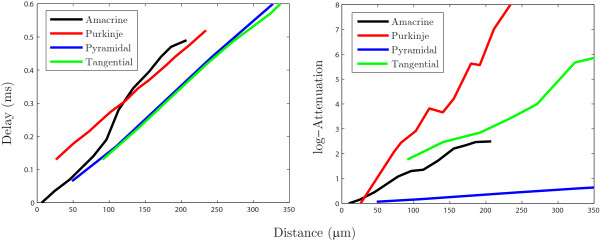
Propagation delay and log-attenuation for reconstructed geometries

**Fig. 9:**
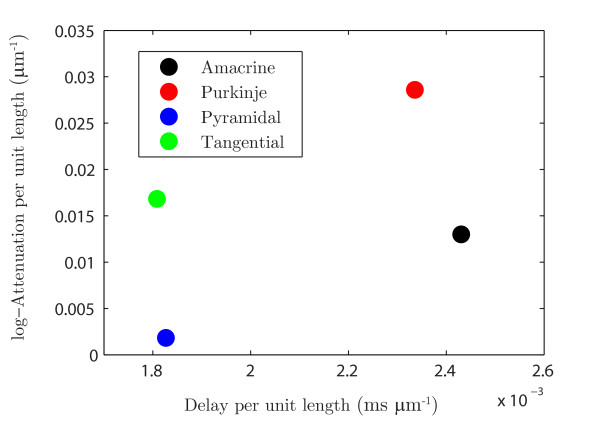
Transfer properties of the response signal for reconstructed geometries

Figure 8 demonstrates the propagation delay and the log-attenuation as a function of distance *y* away from *x* for the reconstructed dendritic morphologies in Figs. 4B–E. The point *x* was placed near the soma as shown in Fig. 4 and the position of *y* was moved away from *x* to the distal dendrites along a single path. As expected with an additive property, both the delay and the log-attenuation are linear in the distance between *y* and *x*. The curves in Fig. 8 show a noisy linear trend, which could be smoothed to better demonstrate this linearity by sampling the data from multiple points located along different branches, but at the same fixed distance away from *x*, in the manner of an expanding sphere of radius *y*, with origin at *x*. To compare how the response signal is transferred in four neuronal types, we plot the rate of change of the delay and log-attenuation for individual cells in Fig. 9, computed by a linear regression and imposing that the line passes through the origin. It succinctly illustrates that the input signal will spread differently in these four cells, with the tangential cell having a similar rate of delay with the pyramidal cell and a similar rate of log-attenuation with the amacrine cell. The signal in the Purkinje cell is shown to be attenuated most, whereas the pyramidal cell transfers it very effectively. Similar conclusions, but about the propagation of the dendritic action potential, were made by Vetter et al. [30]. The activation of voltage-gated channels is expected to be less robust in the case of strong attenuation of the passive spread of voltage which might explain the results in [30]. 

## 5 Discussion

 In this paper, we introduced a number of efficient algorithms for the computational realisation of the sum-over-trips framework and assessed their convergence. We started with some modifications of the four-classes algorithm of Cao and Abbott [21] to avoid constructing duplicate trips. An unambiguous context-free grammar was derived, which is able to generate all trips uniquely and in monotonic order of length. We then developed the length-priority method, in which trips are constructed purely in length order rather than in classes. Both methods were found to demonstrate very nonuniform convergence which was highly-dependent on the dendritic morphology, as well as the biophysical properties of the cell membrane. Oscillations of the convergence error make it difficult to predict the number of trips required for constructing the Green’s function on a particular geometry. Dendritic structures with longer branches of uniform diameters will converge faster, that is, for a smaller number of trips in the series solution. Instead of sampling the trips in some well-defined order, we also derived a stochastic method of sampling the trips based on a Monte-Carlo approach. Finally, we proposed an extremely efficient matrix method which computes the trip coefficients, $A_{\mathrm{trip}}$, for trees where all branches are integer-multiples in length to some base length, Δ*x*.

 Although we considered dendrites to be passive in this study, the proposed algorithms can be easily generalised to support quasi-active (resonant) dendrites with a calculation of the Green’s function in the Laplace domain [24]. Moreover, it is straightforward to include an isopotential soma in the Laplace-domain series solution. A soma can be considered as a special node with the factors $p_{k}(\omega )$ on the branches connected to the soma defined as $p_{k}(\omega )=r_{k}^{-1}\sqrt{(\omega +\tau^{-1})D_{k}^{-1}}/(\hat{C}\omega +{\hat{R}}^{-1}+\sum_{m}r_{m}^{-1}\sqrt{(\omega +\tau^{-1})D_{m}^{-1}})$, where $\hat{C}$ and $\hat{R}$ are the capacitance and the resistance of the somatic membrane and *ω* is the Laplace transform’s frequency variable. Similar factors can also be found for when the leaky-end boundary condition, referred to as *natural termination* by Tuckwell [33], is imposed at the terminals. A knowledge of the Green’s function for a given dendritic structure allows one to efficiently find the sub-threshold voltage response along the entire tree for any number of various inputs, either analytically or via a computation of the convolution integral. This obviates the need for the brute-force numerical simulations of an underlining set of PDEs. Such simulations may be computationally expensive, particularly since they have to be re-initiated each time a new stimulus is introduced. In the case of supra-threshold inputs, which can activate voltage-gated channels known to be present in dendrites of many neurons, the Spike-Diffuse-Spike (SDS) type model [34,35] can be utilised for analysing the propagations of dendritic action potentials. Although the voltage-gated channels in the SDS framework are modelled by piecewise linear instead of nonlinear dynamics, it has been shown that the speed of a wave propagation in the SDS model is in excellent agreement with a more biophysically realistic nonlinear model [36]. However, an analytically tractable SDS model combined with a fast algorithm for constructing the Green’s function on real geometries provides a computationally efficient framework for studying wave scattering in dendrites. 

 Although networks of spatially-extended neural cells can be numerically simulated, there are currently few mathematical studies of such networks. A natural extension might be to consider a network of branched neurons coupled by gap-junctions. The sum-over-trips formalism can then be generalised to support a presence of new boundary conditions. Recent results of Harris and Timofeeva [37] can be applied to the case of tip-to-tip coupling of the dendritic branches. The proposed algorithms can then be modified by including additional sum-over-trips rules. It is worth mentioning that the computational schemes presented in this paper are able to handle cyclic graphs, which may form as a result of gap-junction coupling across several neurons. While the matrix method is expected to be the most efficient for a network of symmetric or regular structures, realistic reconstructions of discretised trees remain within computational reach. For example, given a sparse random matrix, of correct density and of size 20,000, equivalent to a dendritic tree with 10,000 edges, the calculation of $G_{ij}(x,y,t)$ for all *j* up to $k_{\max}=10^{5}$ only takes fifteen seconds on a desktop computer. For comparison, the Purkinje cell reconstruction in Fig. 4D has just under 5,000 branches. For the case of very large, complex irregular structures it might be possible to employ a recently developed technique of reducing the complexity of large dendrites [38] before applying the sum-over-trips methodology. 

## Appendix:  Mathematical Convergence of the Sum-over-trips Series Solution

Here, we consider an identical diffusion coefficient *D* for all branches, although it is possible to generalise this proof to support different diffusion coefficients. Fixing *t* throughout, we let

$$G_{ij}(x,y)=\sum_{\mathrm{trips}}A_{\mathrm{trip}}G_{\infty }(L_{\mathrm{trip}})=\sum_{k=0}^{\infty }\sum_{\begin{array}{c} paths\;with\\ k\;\mathrm{nodes} \end{array}}A_{\mathrm{trip}}G_{\infty }(L_{\mathrm{trip}}),$$

where

(20) $$G_{\infty }(L_{\mathrm{trip}})=\frac{1}{\sqrt{4\pi tD}}e^{-L_{\mathrm{trip}}^{2}/(4Dt)}e^{-t/\tau }.$$

$A_{\mathrm{trip}}$ is a product of *k* factors 2p∈(0,2) and 2p−1∈(−1,1), where *k* is the number of nodes visited by the trip, for any branch diameters. Then, for a trip touching *k* nodes,

(21) $$|A_{\mathrm{trip}}|\leq 2^{k}.$$

There exists a constant B>0 such that every trip touching *k* nodes satisfies

(22) $$L_{\mathrm{trip}}\geq Bk.$$

This makes *B* the coefficient of the lower bound on trip length in terms of the number of nodes in a trip. Intuitively, *Bk* equals the minimum distance between any two nodes where, for this purpose, we count *x* and *y* as nodes.

Let $F_{k}$ be the number of trips with *k* nodes. Since each node has degree d≤3, then

(23) $$F_{k}\leq 3^{k}.$$

We introduce

$$\Gamma_{k}=\sum_{\begin{array}{c} trips\;with\\ k\;\mathrm{nodes} \end{array}}A_{\mathrm{trip}}G_{\infty }(L_{\mathrm{trip}}).$$

Then

(24) $$\begin{array}{rl} \left|\Gamma_{k}\right| & =|\sum_{\begin{array}{c} trips\;with\\ k\;\mathrm{nodes} \end{array}}A_{\mathrm{trip}}G_{\infty }(L_{\mathrm{trip}})|\\ & \leq \sum_{\begin{array}{c} trips\;with\\ k\;\mathrm{nodes} \end{array}}\left|A_{\mathrm{trip}}G_{\infty }(L_{\mathrm{trip}})\right|\\ & =\sum_{\begin{array}{c} trips\;with\\ k\;\mathrm{nodes} \end{array}}|A_{\mathrm{trip}}|\left|G_{\infty }(L_{\mathrm{trip}})\right|. \end{array}$$

For simplicity, we rewrite the Green’s function (20) as $G_{\infty }(L_{\mathrm{trip}})=Ce^{-EL_{\mathrm{trip}}^{2}}$, where

$$C=\frac{e^{-t/\tau }}{\sqrt{4\pi Dt}}\;\text{and}\;E=\frac{1}{4Dt}.$$

Then using (21)–(24) we get

(25) $$\begin{array}{rl} \left|\Gamma_{k}\right| & \leq \sum_{\begin{array}{c} trips\;with\\ k\;\mathrm{nodes} \end{array}}2^{k}Ce^{-EL_{\mathrm{trip}}^{2}}\\ & \leq \sum_{\begin{array}{c} trips\;with\\ k\;\mathrm{nodes} \end{array}}2^{k}Ce^{-EB^{2}k^{2}}\\ & \leq F_{k}2^{k}Ce^{-EB^{2}k^{2}}\\ & \leq 3^{k}2^{k}Ce^{-EB^{2}k^{2}}\\ & =6^{k}Ce^{-EB^{2}k^{2}}\\ & =Ce^{-k(EB^{2}k-\ln(6))}. \end{array}$$

We define $N=\lfloor \ln(6)/(EB^{2})\rfloor$ such that $EB^{2}k-\ln(6)>0$ for ∀k>N. Then

(26) $$\begin{array}{rl} \left|\sum_{k=0}^{\infty }\Gamma_{k}\right| & \leq \sum_{k=0}^{\infty }|\Gamma_{k}|\\ & \leq \sum_{k=0}^{\infty }Ce^{-k(EB^{2}k-\ln(6))}\\ & =\sum_{k=0}^{N}Ce^{-k(EB^{2}k-\ln(6))}+\sum_{k=N+1}^{\infty }Ce^{-k(EB^{2}k-\ln(6))}. \end{array}$$

The first sum in (26) is a finite sum of finite terms, and is hence finite. We will now show that the second sum is also finite using d’Alembert’s ratio criterion for convergent series. The ratio $\rho_{k}$ of the consecutive terms in the series, *k* and k+1, is

$$\begin{array}{rl} \rho_{k} & =\left|\frac{Ce^{-(k+1)(EB^{2}(k+1)-\ln(6))}}{Ce^{-k(EB^{2}k-\ln(6))}}\right|\\ & =e^{-(EB^{2}(2k+1)-\ln(6))}. \end{array}$$

Letting k→∞, we obtain

$$\begin{array}{rl} \rho_{\infty } & =\lim_{k\to \infty }\rho_{k}\\ & =\lim_{k\to \infty }e^{-(EB^{2}(2k+1)-\ln(6))}\\ & =0. \end{array}$$

With $\rho_{\infty }<1$, the second sum in (26) converges absolutely for all constants B,C,E>0. Therefore, the series in (26) is absolutely convergent for sufficiently-high *k*.

If we define

$$G_{ij}^{M}(x,y,t)=\sum_{k=0}^{M}\sum_{\begin{array}{c} trips\;with\\ k\;\mathrm{nodes} \end{array}}A_{\mathrm{trip}}G_{\infty }(L_{\mathrm{trip}},t),$$

then

$$|G_{ij}-G_{ij}^{M}|\leq \sum_{k=M+1}^{\infty }Ce^{-k(EB^{2}k-\ln(6))}$$

and the path integral converges faster than $e^{-k}$ in the worst case, with the number of nodes *k* visited by the trips.

## Competing interests

The authors declare that they have no competing interests.

## Authors’ contributions

QC was directly involved in developing and implementing the algorithms, carried out all analysis, and drafted the manuscript. SRD participated in developing and implementing the algorithms. SPBC developed the Monte-Carlo algorithm. YT conceived and guided the study. All authors contributed improvements to the final manuscript, which they have read and approved.
